# SFTPA1 is a potential prognostic biomarker correlated with immune cell infiltration and response to immunotherapy in lung adenocarcinoma

**DOI:** 10.1007/s00262-021-02995-4

**Published:** 2021-06-28

**Authors:** Lu Yuan, Xixi Wu, Longshan Zhang, Mi Yang, Xiaoqing Wang, Wenqi Huang, Hua Pan, Yuting Wu, Jihong Huang, Wenyu Liang, Jiaxin Li, Xiaodi Zhu, Shuang Wang, Jian Guan, Laiyu Liu

**Affiliations:** 1 Chronic Airways Diseases Laboratory, Department of Respiratory and Critical Care Medicine, Nanfang Hospital, Southern Medical University, Guangzhou, Guangdong China; 2 Department of Radiation Oncology, Nanfang Hospital, Southern Medical University, Guangzhou, Guangdong China; 3 Department of Pathology, Nanfang Hospital, Southern Medical University, Guangzhou, 510515 China; 4 Department of Pathology, School of Basic Medical Sciences, Southern Medical University, Guangzhou, China

**Keywords:** Surfactant protein A1, Prognosis, Immune infiltration, Immunotherapy, Bioinformatics analysis

## Abstract

Pulmonary surfactant protein A1 (SFTPA1) is a member of the C-type lectin subfamily that plays a critical role in maintaining lung tissue homeostasis and the innate immune response. SFTPA1 disruption can cause several acute or chronic lung diseases, including lung cancer. However, little research has been performed to associate SFTPA1 with immune cell infiltration and the response to immunotherapy in lung cancer. The findings of our study describe the SFTPA1 expression profile in multiple databases and was validated in BALB/c mice, human tumor tissues, and paired normal tissues using an immunohistochemistry assay. High SFTPA1 mRNA expression was associated with a favorable prognosis through a survival analysis in lung adenocarcinoma (LUAD) samples from TCGA. Further GeneOntology (GO) and Kyoto Encyclopedia of Genes and Genomes (KEGG) pathway enrichment analyses showed that SFTPA1 was involved in the toll-like receptor signaling pathway. An immune infiltration analysis clarified that high SFTPA1 expression was associated with an increased number of M1 macrophages, CD8^+^ T cells, memory activated CD4^+^ T cells, regulatory T cells, as well as a reduced number of M2 macrophages. Our clinical data suggest that SFTPA1 may serve as a biomarker for predicting a favorable response to immunotherapy for patients with LUAD. Collectively, our study extends the expression profile and potential regulatory pathways of SFTPA1 and may provide a potential biomarker for establishing novel preventive and therapeutic strategies for lung adenocarcinoma.

**Supplementary Information:**

The online version contains supplementary material available at 10.1007/s00262-021-02995-4.

## Introduction

Lung cancer remains the leading cause of cancer incidence and mortality. According to the latest GlOBOCAN 2018 data, there are approximately 2.1 million new cases of lung cancer cases in the word in 2018, and about 1.8 million people died from lung cancer, representing nearly one-fifth (18.4%) of cancer-related death [1]. Since 2015, immune checkpoint inhibitors have been approved as either monotherapy or for use in combination with other agents for the treatment of NSCLC [2]. An immune checkpoint blockade (ICB) functions by blocking the receptor or the ligand interactions of molecules (e.g., CTLA-4 and PD-1), which provides novel hope for lung cancer patients with negative driver oncogenes or resistance to molecular-targeted therapy. However, the lack of an accurate prediction of dominant populations represents a major bottleneck in cancer immunotherapy. Therefore, there is a requirement to develop additional biomarkers to ensure that larger patient populations will benefit.

Recently, the tumor immune microenvironment (TIME) has been shown to strongly influence cancer growth and the response to immunotherapy [3, 4]. A previous study demonstrated that an IL-6 blockade can limit the development and progression of K-ras-Mutant lung cancer by altering the relative proportion between protumor and antitumor immune cells [5]. Gene alterations have also been recently reported to be an important predictor of the response to immunotherapy [6–8]. For example, Ronald et al. found that a deletion of tumor suppressor gene, PTEN, could activate the YAP1-LOX axis to recruit tumor-related macrophages, and infiltrating macrophages secreted SPP1 to support tumor cell growth and stimulate angiogenesis in glioblastoma multiforme [9]. Based on these reports, we sought to identify a novel biomarker for immunotherapy in lung cancer.

SFTPA1 is a pulmonary surface-active substance synthesized by type II alveolar epithelial cells. In this study, we sought to characterize the expression, regulatory mechanism, and clinical value of SFTPA1 using an integrated bioinformatics analysis.

## Materials and methods

### Human tissue samples

A total of 66 cases of human paired tumor and adjacent normal tissues were collected from cancer patients who underwent surgical resection at Nanfang Hospital (Guangzhou, China) from January 2016 to June 2019, including patients with bladder urothelial carcinoma (BLCA), breast invasive carcinoma (BRCA), cervical squamous cell carcinoma (CESC), colon adenocarcinoma (COAD), esophageal carcinoma (ESCA), head and neck squamous cell carcinoma (HNSC), kidney renal clear cell carcinoma (KIRC), liver hepatocellular carcinoma (LIHC), lung adenocarcinoma (LUAD), lung squamous cell carcinoma (LUSC), prostate adenocarcinoma (PRAD), rectum adenocarcinoma (READ), skin cutaneous melanoma (SKCM), stomach adenocarcinoma (STAD), and thyroid carcinoma (THCA). No treatment was administered prior to surgery. At least three patients were included for each type of cancer. The 12 lung cancer tissues from patients treated with pembrolizumab-based therapy were collected prior to receiving immunotherapy at Nanfang Hospital (Guangzhou, China) from May 2018 to August 2020. The collection of human tissue samples was approved by the Ethics Committee of Nanfang Hospital.

### Mice tissue samples

A total of six male BALB/c mice (4–6-weeks-old; weighing 18–22 g) were obtained from the Central Laboratory of Animal Science at Southern University (Guangzhou, China). All experiments were approved by the Institutional Animal Care and Use Committee in Southern Medical University and conformed with the guidelines for the use of laboratory animals. The BALB/c mice had free access to food and water in specific pathogen-free (SPF)-grade housing of the laboratory. The mice were sacrificed by cervical dislocation, and the tissues were obtained from different organs.

### Immunohistochemistry (IHC) assay

Immunohistochemistry (IHC) was performed to determine the expression and subcellular locations of SFTPA1 in human normal tissues, paired tumors tissues, and BALB/c mice tissues. All sections were incubated at 65 °C for 1.5 h, deparaffinized in xylene, and rehydrated in graded ethanol. Antigen retrieval was performed in ETDA buffer (pH 9.0) and boiled at 100 °C for 5 min. Endogenous peroxidase activity was eliminated with 3% H_2_O_2_ for 15 min. The sections were incubated with a rabbit anti-SFTPA1 primary antibody (1:100, Proteintech, 11,850–1-AP) at 4℃ overnight. The goat anti-rabbit secondary antibody was incubated with the sections for 45 min at 37℃. The washed sections were visualized using 3,3-diaminobenzidine (DAB). The DAB-stained sections were counterstained in hematoxylin, dehydrated in graded ethanol, sealed with neutral balsam, and observed under a microscope. The percentage of SFTPA1-positive cells and staining intensity were individually estimated in five random fields per section by two independent observers. The expression of SFTPA1 was calculated for both the proportion of positively stained tumor cells and the staining intensity. The proportion of positivity was scored from 0 to 4 as follows: “0”, 0%; “1”, 1% – 25%; “2” 26% – 50%; “3” 51% – 75%; and (4) > 75%. The staining intensity was scored from 0 to 3 as follows: “0” negative staining; “1” weak staining; “2” moderate staining; and “3” strong staining. The staining was assessed as follows: a final staining score of < 3 was considered to be “negative”; a final staining score of 3 was regarded as “weak”; a final staining score of 4 was assessed as “moderate”, and a final staining score of ≥ 5 was considered to be “strong”[10].

### Survival analysis

Survival curves were described according to the level of SFTPA1 mRNA expression in the Pan-cancer RNA-sequence data derived from a Kaplan–Meier Plotter Database (http://kmplot.com/analysis/), an online tool that incorporates the public microarray data from the GEO and TCGA databases. The patients were divided into high and low expression groups using the auto-select best cut-off. Two other independent LUAD data sets from the GEO database (GSE13213 and GSE42127) were used as validation. The median value of SFTPA1 mRNA expression was used as the cut-off value. A threshold of P < 0.05 was considered significant.

### Protein–protein interaction (PPI) network and functional annotation analysis

A PPI network of SFTPA1 co-expression genes was constructed using the STRING database (https://string-db.org/) and visualized with Cytoscape software (Version 3.7.1). The gene set involved in the PPI network was uploaded to the Database for Annotation, Visualization, and Integrated Discovery (DAVID Version 6.8), a functional annotation tool (https://david.ncifcrf.gov/summary.jsp) for analyzing the Gene Ontology (GO) and Kyoto Encyclopedia of Genes and Genomes (KEGG) pathways. FDR < 0.05 was considered significant.

### Gene set enrichment (GSEA) analysis

The GSEA analysis was performed using GSEA Version 4.1.0. We classified the LUAD tumor samples from the TCGA database into two groups based on the level of SFTPA1 mRNA expression (the median value of SFTPA1 mRNA was used as the cut-off value) and identified the biological pathways that were significantly enriched in the gene set. When performing a GSEA analysis, ic2.cp.kegg.v7.0.symbols.gmt was used as the background gene set, which was downloaded from the MSigDB database found on the GSEA website. The following parameters were used: number of permutations = 1000, permutation type = phenotype. Other parameters were left as the default values. A series of gene sets were selected based on a standard of FDR < 0.05.

### Immune infiltration analysis

The ESTIMATE (http://bioinformatics.mdanderson.org/estimate/) database uses the unique properties of tumor sample transcription profiles to infer the tumor cell density and different infiltrating normal cells [11]. We explored the level of stromal cells and the level of immune cell infiltration in the tumor tissues. CIBERSORT (http://cibersort.stanford.edu/) is a tool for deconvolution of the expression matrix of immune cell subtypes based on the principle of linear support vector regression [12]. TISIDB (http://cis.hku.hk/TISIDB/) is a web portal for tumor and immune system interactions, which integrates multiple heterogeneous data types [13].

The ESTIMATE database was used to explore the relationship between the immune-associated score, clinical features, and SFTPA1 mRNA expression. CIBERSORT and TISIDB were used to analyse the different levels of immune cell infiltration associated with SFTPA1 mRNA expression.

### ceRNAs network

MicroRNAs (miRNAs) were predicted using Targetscan (http://www.targetscan.org/), mirDIP (http://ophid.utoronto.ca/mirDIP/index.jsp), mirCode (http://www.mircode.org) databases, and up-regulated miRNAs in the TCGA LUAD tumor samples. A Venn diagram was constructed using candidate lncRNAs that likely target SFTPA1 using DIANA and down-regulated lncRNAs in the TCGA LUAD tumor samples. The ceRNA network was visualized using Cytoscape software.

### Statistical analysis

All data analyses were performed using SPSS19.0 (IBM, USA) and GraphPad 8.0 software. A Student’s t-test was applied for comparisons between the two groups. The association between the variables was assessed by a Spearman correlation analysis. A P-value < 0.05 was considered to be statistically significant.

## Results

### The expression profile of SFTPA1 in humans

**Fig. 1:**
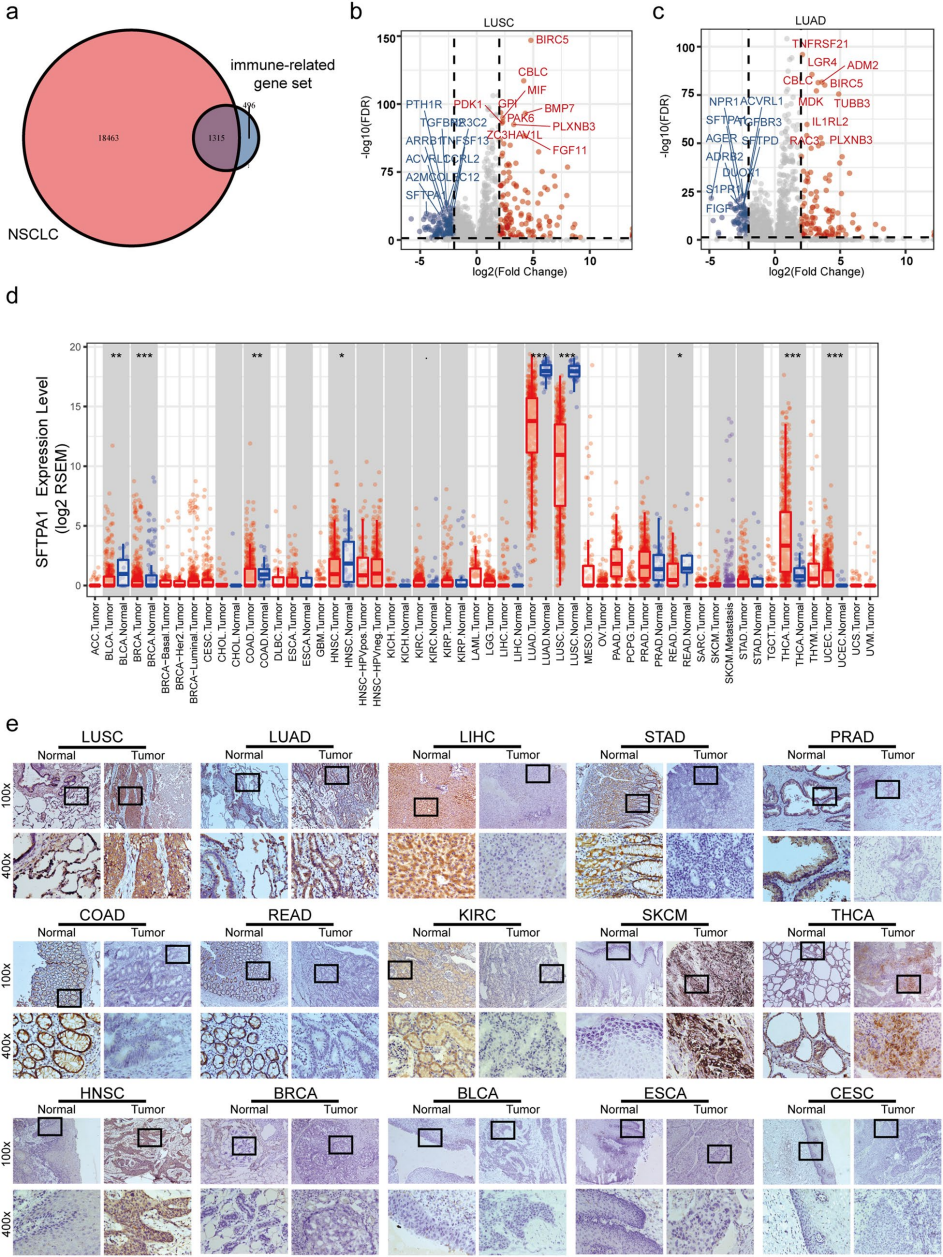
Process of screening the target gene and SFTPA1 expression pattern in human tissues. **a** Venn diagram of genes selected by the NSCLC gene set from the TCGA database and immune gene set from ImmPort. **b** & **c** Volcano plot of the differential gene expression in the tumor vs adjacent normal comparison in LUSC **b** and LUAD **c** (P < 0.01, fold-change > 1). **d** The level of SFTPA1 mRNA expression in various human tumors and adjacent normal tissues in TIMER across all TCGA tumors (P-value Significant Codes: 0 ≤ *** < 0.001 ≤ ** < 0.01 ≤ * < 0.05 ≤ . < 0.1). **e** The SFTPA1 protein expression pattern in 15 human tumors and adjacent normal tissues by immunohistochemistry. BLCA, bladder urothelial carcinoma; BRCA, breast invasive carcinoma; CESC, cervical squamous cell carcinoma; COAD, colon adenocarcinoma; ESCA, esophageal carcinoma; HNSC, head and neck squamous cell carcinoma; KIRC, kidney renal clear cell carcinoma; LIHC, liver hepatocellular carcinoma; LUAD, lung adenocarcinoma; LUSC, lung squamous cell carcinoma; PRAD, prostate adenocarcinoma; READ, rectum adenocarcinoma; SKCM, skin cutaneous melanoma; STAD, stomach adenocarcinoma; THCA, thyroid carcinoma

A flow chart of the target gene screening process is described in Fig. 1.The intersection between the NSCLC gene dataset from the Cancer Genome Atlas (TCGA) database and immune gene dataset from ImmPort [14] obtained 1315 immune genes (Fig. 1a). A differential expression analysis of these genes in LUSC and LUAD tumor and normal tissues was performed (P < 0.01, fold-change > 1) (Fig. 1b and c). Finally, SFTPA1, PLXNB3, BIRC5, CBLC, and ACVRL1 were screened out based on the intersection between the top 10 differentially expressed genes in lung adenocarcinoma (LUAD) and lung squamous cell carcinoma (LUSC) (Supplementary Fig. 1a). We then focused on surfactant protein A1 (SFTPA1) based on the highest level of expression, which should aid in further experimental verification.

The GTExPOrtal database was used to explore the level of SFTPA1 expression in normal human tissues. SFTPA1 was primarily expressed in the lung tissues and moderately expressed in other tissues (Supplementary Fig. 1b). According to the Oncomine online database, the downregulation of SFTPA1 expression was observed in the brain and central nervous system cancer, breast cancer, and lung cancer, whereas it was up-regulated in prostate cancer (Supplementary Fig. 1c). We further detected SFTPA1 mRNA expression in various human tumors and adjacent normal tissues in TIMER across all TCGA tumors. Consistent with the results in normal human tissues, SFTPA1 mRNA was primarily expressed in lung cancer. The expression of SFTPA1 mRNA was lower in LUAD, LUSC, BLCA, BRCA, COAD, HNSC, and READ compared to the corresponding normal tissues, whereas it was higher in THCA and UCEC (Fig. 1d; P < 0.05).

The immunohistochemical results of the human tumor tissues (tumor) and paired adjacent normal tissues (normal) from patients with 15 different types of cancers are shown in Fig. 1e. We found that the SFTPA1 protein was strongly expressed in both tumors and the corresponding normal tissues in LUSC and LUAD, which is consistent with the TIMER results. Both the tumor and adjacent normal tissues, negative staining was detected in BRCA, BLCA, ESCA, and CESC. Compared with the tumor tissues, moderate to strong positive staining was observed in the adjacent noncancerous tissue of LIHC, STAD, PRAD, COAD, READ, and KIRC; however, contrasting results were obtained in SKCM, THCA, and HNSC, which were positively expressed in the tumor tissues.

### The expression profile of SFTPA1 in BALB/c mice

**Fig. 2:**
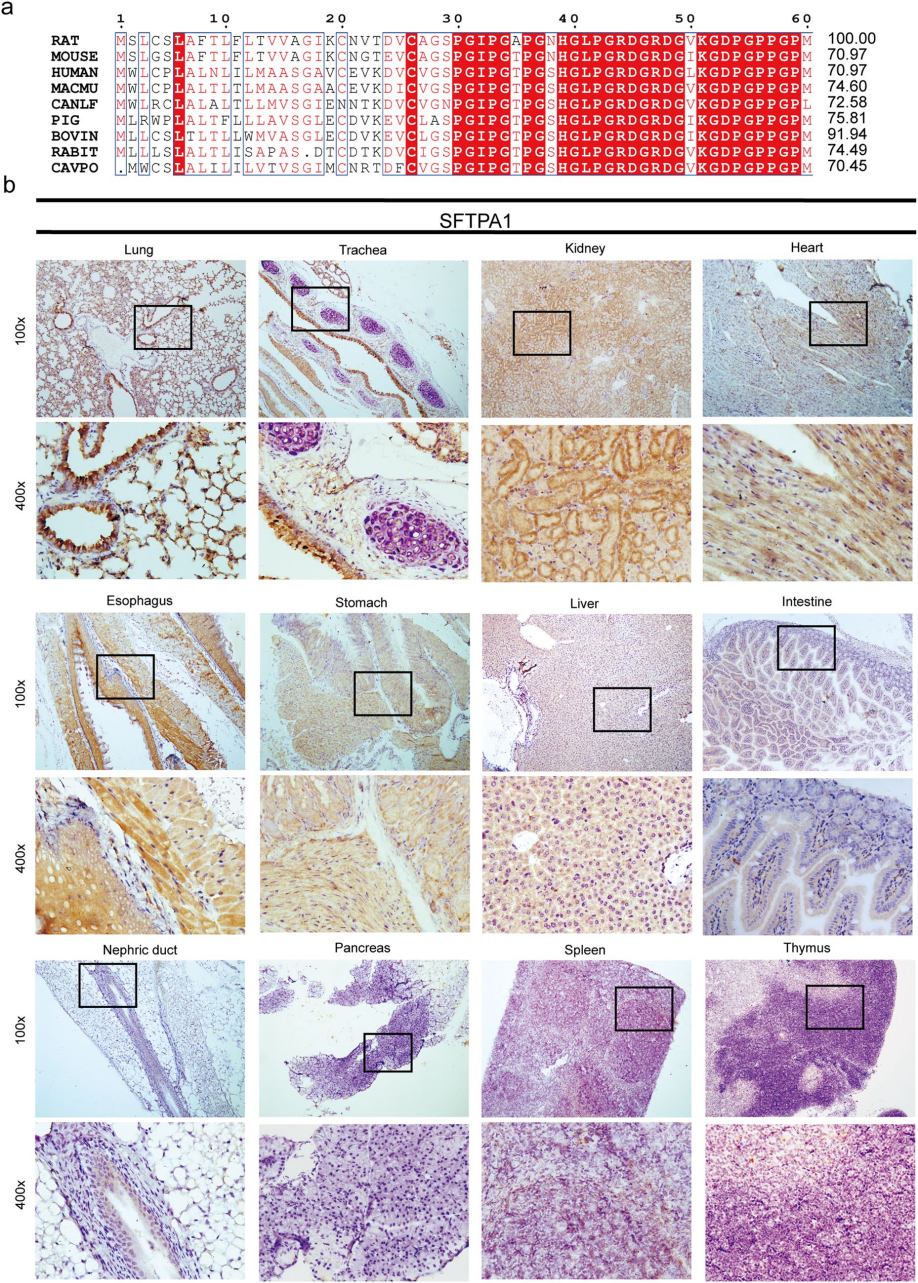
Protein homology analysis and SFTPA1 expression pattern in mammalian tissue tissues. **a** Alignment of the SFTPA1 protein sequences between human and rat, mouse, macmu (macaca mulatta), canlf (dog), pig, bovine, rabbit, and cavpo (guinea pig). The alignment scores are shown on the right. **b** SFTPA1 protein expression patterns in various tissues of BALB/c mice by immunohistochemistry

A protein homology analysis revealed that human SFTPA1 shared 70.97%, 70.97%,74.60%, 72.58%, 75∙81%, 91.94%, 74.49%, and 70.45% similarity with rat, mouse, macau (macaca mulatta), canlf (dog), pig, bovin, rabit, and cavpo (guinea pig), respectively (Fig. 2a). This revealed a high level of conservation among these species. Among these, mice are among the most commonly used laboratory animals. To investigate the tissue distribution of SFTPA1 in BALB/c mice, SFTPA1 protein expression was assayed by immunohistochemistry in the different organ tissues (Fig. 2b). In the lung, both epithelial cells of the terminal bronchiole and the alveolar wall displayed strong positive staining in the cytoplasm. In the trachea, the pseudostratified ciliated columnar epithelium exhibited strong staining in the cytoplasm, whereas negative staining was exhibited in the chondrocytes of the hyaline cartilage. In the digestive system, smooth muscle cells and squamous epithelial cells of the esophagus exhibited medium to strong positive staining in the cytoplasm. The cytoplasm of the gastric gland cells and submucosa smooth muscle cells also showed moderate staining. In the intestine, the epithelial cells showed weak positive staining in the cytoplasm. In the liver, weak to moderate positive staining was found in the cytoplasm of hepatocytes. In the urinary system, medium to strong positive staining was evident in the cytoplasm of renal tubular epithelial cells, whereas no staining was observed in glomerular epithelial cells and the nephric ducts. Cardiomyocytes exhibited moderate positive staining, whereas no staining was found in the pancreas, spleen, or thymus tissues.

### Survival analysis of SFTPA1 in lung cancers

**Fig. 3:**
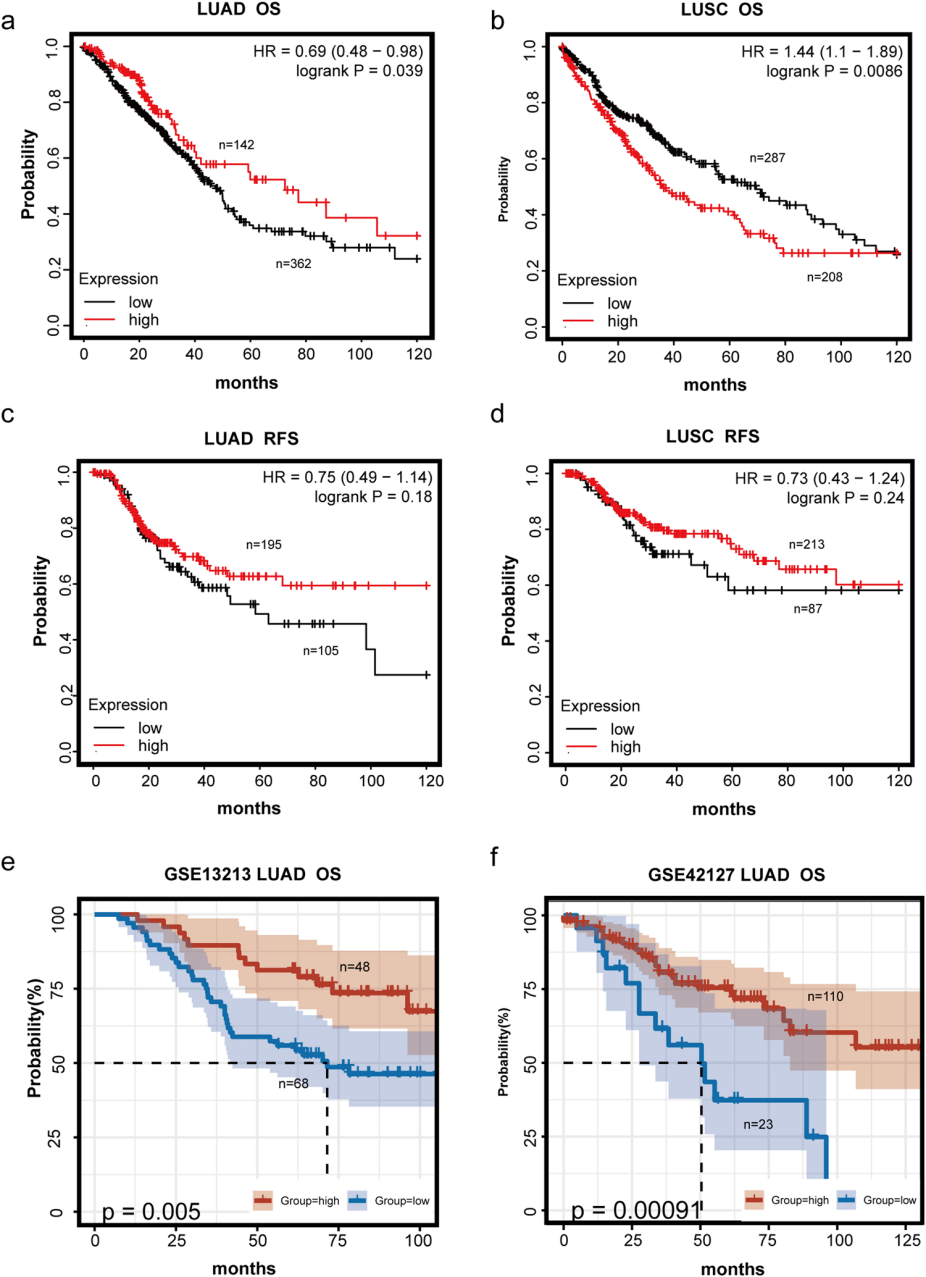
The prognostic value of SFTPA1 in lung cancer. **a** & **b** OS of two different levels of SFTPA1 expression in TCGA LUAD (**a**) and LUSC (**b**). **c** & **d**. RFS of two different levels of SFTPA1 expression in TCGA LUAD (**c**) and LUSC (**d**). **e** & **f**. OS of two different levels of SFTPA1 expression in LUAD from the GEO database (GSE13213 (**e**) and GSE42127 (**f**))

To assess the prognostic value of SFTPA1, survival analyses were subsequently conducted in LUAD and LUSC. Remarkably, the prognostic significance of SFTPA was not consistent in LUSC and LUAD. High SFTPA1 expression was associated with significantly longer overall survival (OS) in LUAD (*P* = 0.039, *HR* = 0.69) (Fig. 3a). Instead, low SFTPA1 expression was associated with a longer OS in LUSC (*P* = 0.0086, *HR* = 1.44) (Fig. 3b). This likely occurred due to the functional heterogeneity of SFTPA1 in different pathological types. The relapse-free survival curve (RFS) showed no significant difference between RFS and SFTPA1 expression, regardless of LUAD or LUSC (*P* > 0.05) (Fig. 3c and d). Two other different datasets from the GEO database implied a similar finding that high SFTPA1 expression was significantly associated with a better OS in LUAD (Fig. 3e and f).

### Functional enrichment analyses of SFTPA1 in LUAD

**Fig. 4:**
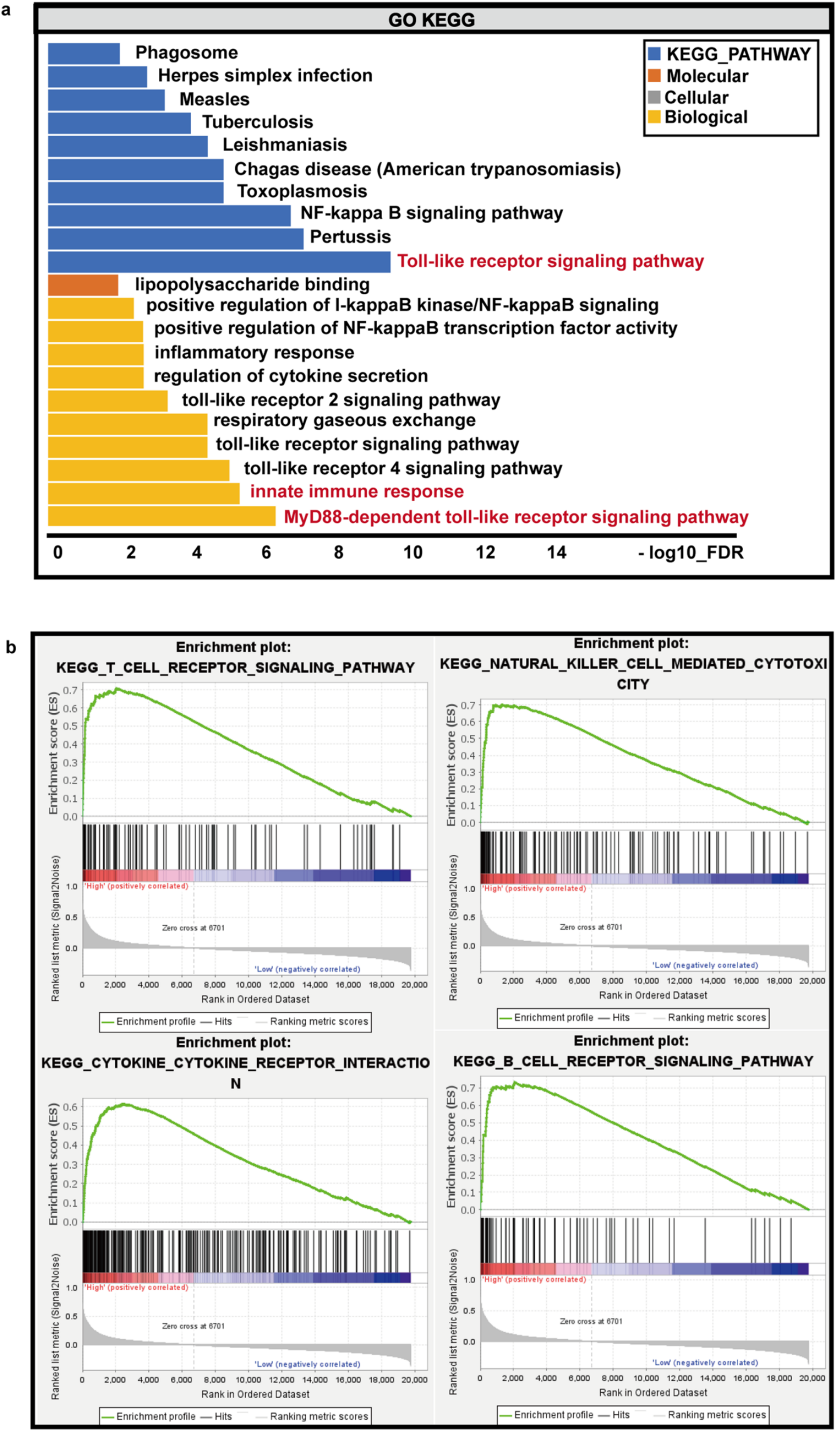
Functional enrichment analysis of SFTPA1 in LUAD. **a** GO and KEGG pathway enrichment analyses of the SFTPA1 co-expression genes. **b** The gene set enrichment analysis (GSEA) results enriched in the SFTPA1-high expression group

We next sought to identify the underlying mechanism of SFTPA1. As shown in Supplementary Fig. 1a, the PPI network was constructed for the co-expression of genes using the STRING database and visualized by Cytoscape. We then performed a GO and KEGG pathway enrichment analyses for these co-expression genes (Fig. 4a). The GO enrichment analysis was composed of biological process (BP), cellular component (CC), and molecular function (MF). Biological process was significantly enriched in the MyD88-dependent toll-like receptor signaling pathway, innate immune response, and toll-like receptor 4 signaling pathway. MF was enriched in lipopolysaccharide binding, and no significant results in CC were enriched after rigorous screening. The KEGG pathway was significantly enriched in the Toll-like receptor signaling pathway. These results revealed that SFTPA1 was closely correlated with the toll-like receptor signaling pathway in the innate immune response.

Since the immune-related pathways were enriched, we next performed a GSEA to further clarify the impact of SFTPA1 in LUAD. A total of 513 lung adenocarcinoma samples from TCGA database were divided into two groups based on the median SFTPA1 expression data, and ic2.cp.kegg.v7.0.symbols.gmt was used as the background gene set. A total of 103 genes were up-regulated, and 75 genes were down-regulated in each of the two groups (Fig. 4b and Supplementary Table 1 and Supplementary Table 2). In the SFTPA1-high group, the top five molecular pathways enriched by GSEA were natural killer cell-mediated cytotoxicity, cell adhesion molecule cams, the T cell receptor signaling pathway, cytokine receptor interaction, and the B cell receptor signaling pathway. These results demonstrate that SFTPA1 may function as a tumor suppressor via modulating the immune system in lung adenocarcinoma.

### The correlation between SFTPA1 expression and the immune infiltration score in LUAD

**Fig. 5:**
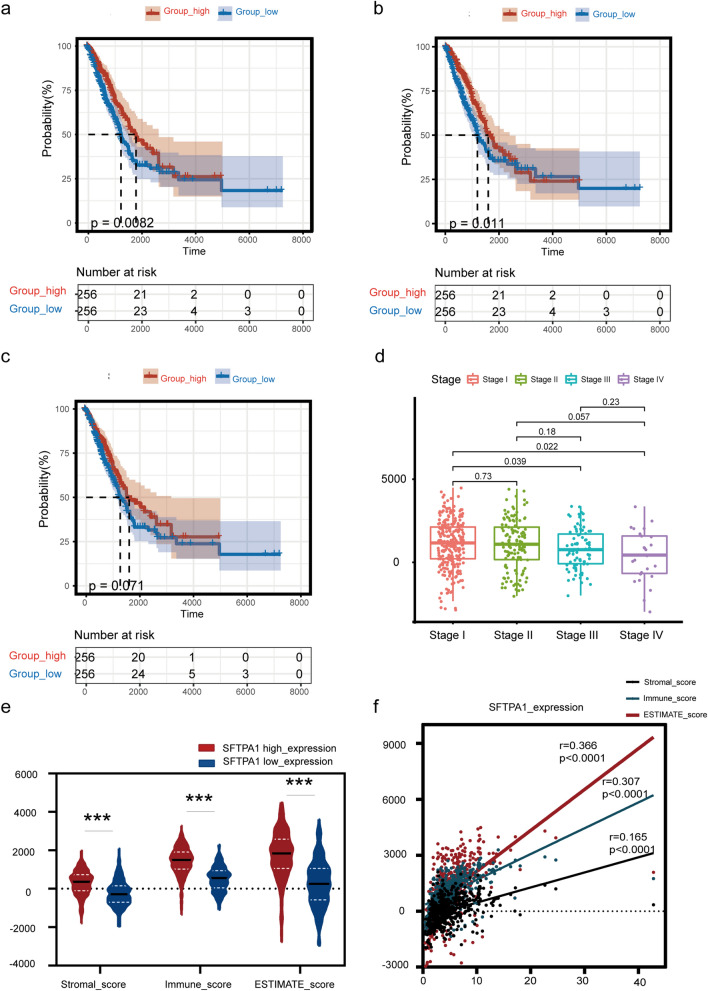
The different immune infiltration scores in the SFTPA1-high/low expression groups. The immune infiltration score, including the immune score, stromal score, and ESTIMATE score. The analysis was conducted using the ESTIMATE database. (P-value Significant Codes: 0 ≤ *** < 0.001 ≤ ** < 0.01 ≤ * < 0.05). **a**–**c**. OS of two different ESTIMATE scores (**a**), immune scores (**b**), and stromal scores (**c**). **d** ESTIMATE score and stage. **e** The immune, stromal, and ESTIMATE scores in the SFTPA1-high expression group (*n* = 256) compared with the SFTPA1-low expression group (*n* = 256). The median is the threshold. **f** Correlation between the level of SFTPA1 expression and immune score, stromal score, and ESTIMATE score

Several studies have demonstrated that SFTPA regulates the adaptive and innate immune cell functions in pulmonary infectious diseases, particularly the function of macrophages [15–17]. To better understand the relevance between SFTPA1 and the immune system in LUAD, we investigated the correlation between SFTPA1 expression and the level of immune infiltration using the ESTIMATE database. Firstly, we found that high ESTIMATE and immune scores were both significantly associated with a longer OS in LUAD (ESTIMATE score: *P* = 0.0082; immune score: *P* = 0.011, Fig. 5a and b), whereas the stromal score was not statistically significant for survival (*P* > 0.05, Fig. 5c). Furthermore, the ESTIMATE score was significantly associated with gender and TNM stage in patients with LUAD (*P* < 0.05, Fig. 5d). A total of 513 TCGA LUAD samples were divided into high/low expression groups based on the median SFTPA1 expression data. Statistical differences were found in the ESTIMATE, immune, and stromal scores between the two groups (Fig. 5e). A Spearman correlation analysis showed that SFTPA1 expression was positively correlated with the ESTIMATE, immune, and stromal scores (*P* < 0.0001; ESTIMATE score: *r* = 0.366; immune score: r = 0.307; stromal score *r* = 0.165; Fig. 5f).

### The differential immune cell infiltration landscape in the SFTPA1-high/low expression groups

**Fig. 6:**
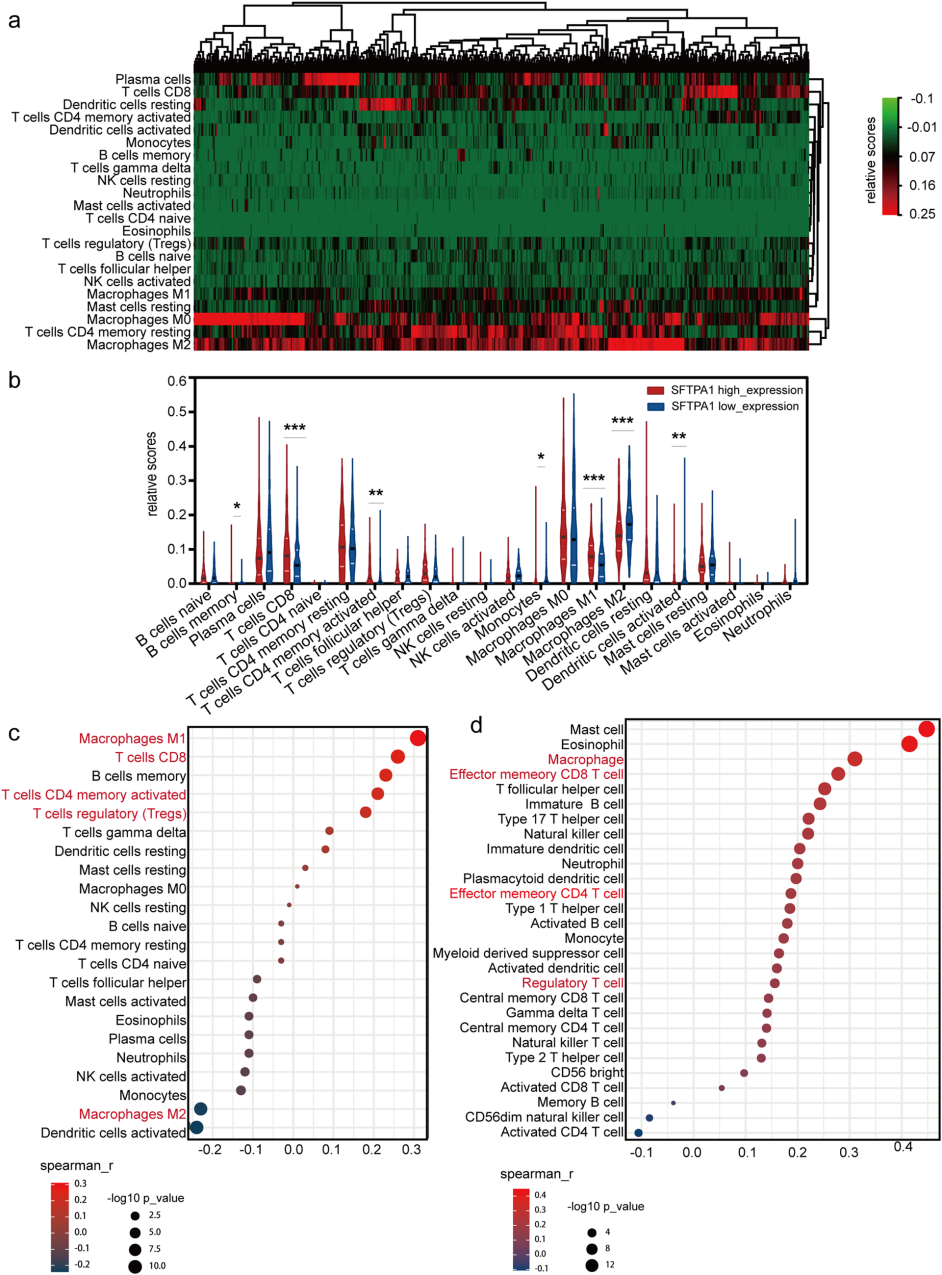
Immune cell infiltration landscape in SFTPA1-high/low expression groups in LUAD **a** Heatmap of 22 immune cell subpopulations based on deconvolution by CIBERSORT in LUAD. **b** There were 22 immune cell subpopulations in the SFTPA1-high expression group compared with the SFTPA1-low expression group in LUAD. (t-test, P-value Significant Codes: 0 ≤ *** < 0.001 ≤ ** < 0.01 ≤ * < 0.05). **c** & **d** Spearman correlation between the level of SFTPA1 expression and immune cell subpopulations by CIBERSORT (**c**) and TISIDB (**d**)

As mentioned above, SFTPA1 was involved in the immune response and was associated with the immune infiltration score. Moreover, a previous study reported that SFTPA1 inhibited lung cancer progression by controlling M1 macrophage polarization in mice models [18]. To further explore the relationship between SFTPA1 and tumor immune cell infiltration landscape, we utilized the CIBERSORT algorithm to first calculate the relative proportion of 22 types of immune cells in the TCGA LUAD (Fig. 6a). This cohort displayed a greater portion of M0 macrophages, CD4^+^ memory resting T cells, M2 macrophages, dendritic cells, as well as resting and CD8^+^ T cells. Additionally, seven types of immune cells exhibited statistically significant differences between the SFTPA1 high and low expression group, including B cells, memory T cells, CD8^+^ T cells, CD4^+^ activated memory cells, monocytes, M1 macrophages, M2 macrophages, and activated dendritic cells (Fig. 6b). Unlike LUAD, the high expression of SFTPA1 was associated with resting mast cells in LUSC (Supplementary Fig. 2b). Immediately afterward, a Spearman correlation analysis was performed with CIBERSORT (Fig. 6c) and TISIDB (Fig. 6d). Combining the results of these two methods, it was concluded that M1 macrophages, CD8^+^ T cells, activated memory CD4^+^ T cells, and regulatory T cells were significantly positively correlated with SFTPA1 expression. In contrast, M2 macrophages were significantly negatively correlated with SFTPA1 expression. Next, the top 10 immune genes with the strongest correlation with SFTPA1 were screened in LUAD, including SFTPA2, SFTPD, AGER, AGTR2, DMBT1, RXRG, VIPR1, SERPIND1, DUOX1, and TMEM173 (Supplementary Fig. 2c and Supplementary Table 3). A Kaplan–Meier analysis revealed that high expression of eight genes in the above genes was correlated with the OS in LUAD (Supplementary Fig. 2d). A heatmap of the correlation between SFTPA1-related genes and the immune cell infiltration landscape is shown in Supplementary Fig. 2e. These results showed that SFTPA1 could promote the formation of an active immune microenvironment in LUAD.

### CeRNA network of SFTPA1 in LUAD

**Fig. 7:**
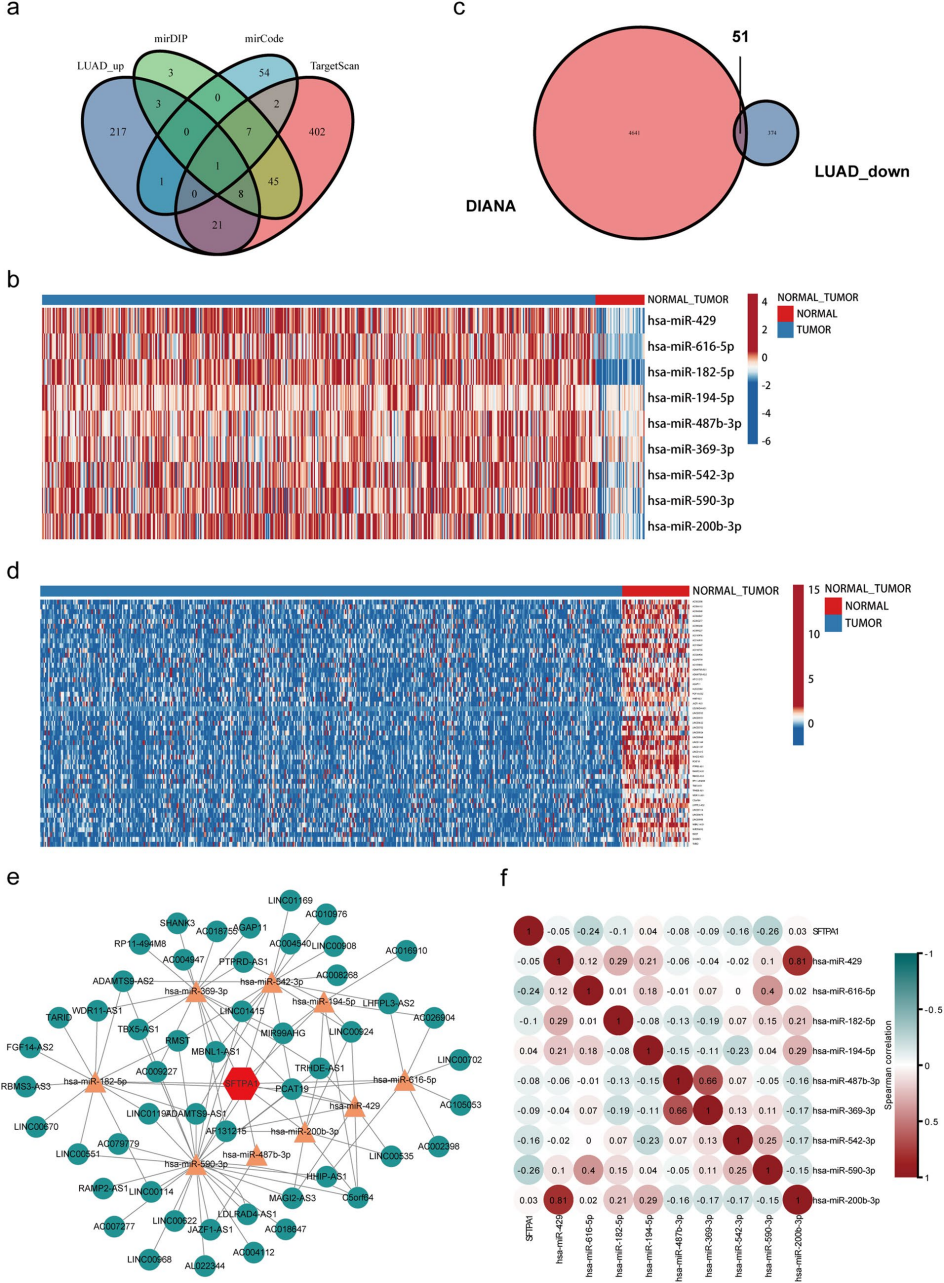
The ceRNA network of SFTPA1 in TCGA LUAD. **a** Venn diagram of the candidate miRNAs targeting SFTPA1 using Targetscan, miRwalk, mirRDB databases, and up-regulated miRNAs in the TCGA LUAD. **b** & **d**. Heatmap of the mRNA expression of nine predicted miRNAs (**b**) and 51 lncRNAs (**d**) between normal tissues and tumors. **c** Venn diagram of the candidate lncRNAs that likely target SFTPA1 using DIANA and down-regulating lncRNAs in TCGA LUAD. **e** Construction of the lncRNA-miRNA-SFTPA1 network. **f** Heatmap of the relevance of SFTPA1 expression and nine candidate miRNAs

Our results showed that SFTPA1 was down-regulated in the tumor tissues compared with the normal tissues in LUAD. We next sought to determine the mechanism of SFTPA1 downregulation. It is known to all that microRNAs (miRNAs) play an important role in the regulation of gene expression due to negative post-transcription regulators. Recently, the competing endogenous RNA (ceRNA) hypothesis was considered to be a novel mechanism. CeRNAs could eliminate the inhibition of miRNA on their target genes by competitively binding to the same miRNA response elements [19]. Therefore, a ceRNA-miRNA-mRNA network may reveal the mechanism of SFTPA1 downregulation. First, nine miRNAs targeted to SFTPA1 were predicted using the Targetscan, miRwalk, and mirRDB databases, as well as the up-regulated miRNAs in TCGA LUAD (Fig. 7a). A heat map revealed that the level of candidate miRNA expression between normal and tumor tissues in TCGA LUAD (Fig. 7b). Next, we predicted 4641 lncRNAs based on the interactions of these miRNAs using DIANA. In TCGA LUAD, 374 lncRNAs were down-regulated in the tumor tissues. Therefore, we identified 51 lncRNAs by taking the intersection of DIANA and TCGA LUAD set (Fig. 7c and d). Ultimately, a lncRNA-miRNA-SFTPA1 ceRNA network was constructed in LUAD (Fig. 7e). The Spearman correlation analysis revealed a correlation between has-miR-590-3p and SFTPA1 expression (Fig. 7f).

### The predictive value of SFTPA1 in the response to immunotherapy

**Fig. 8:**
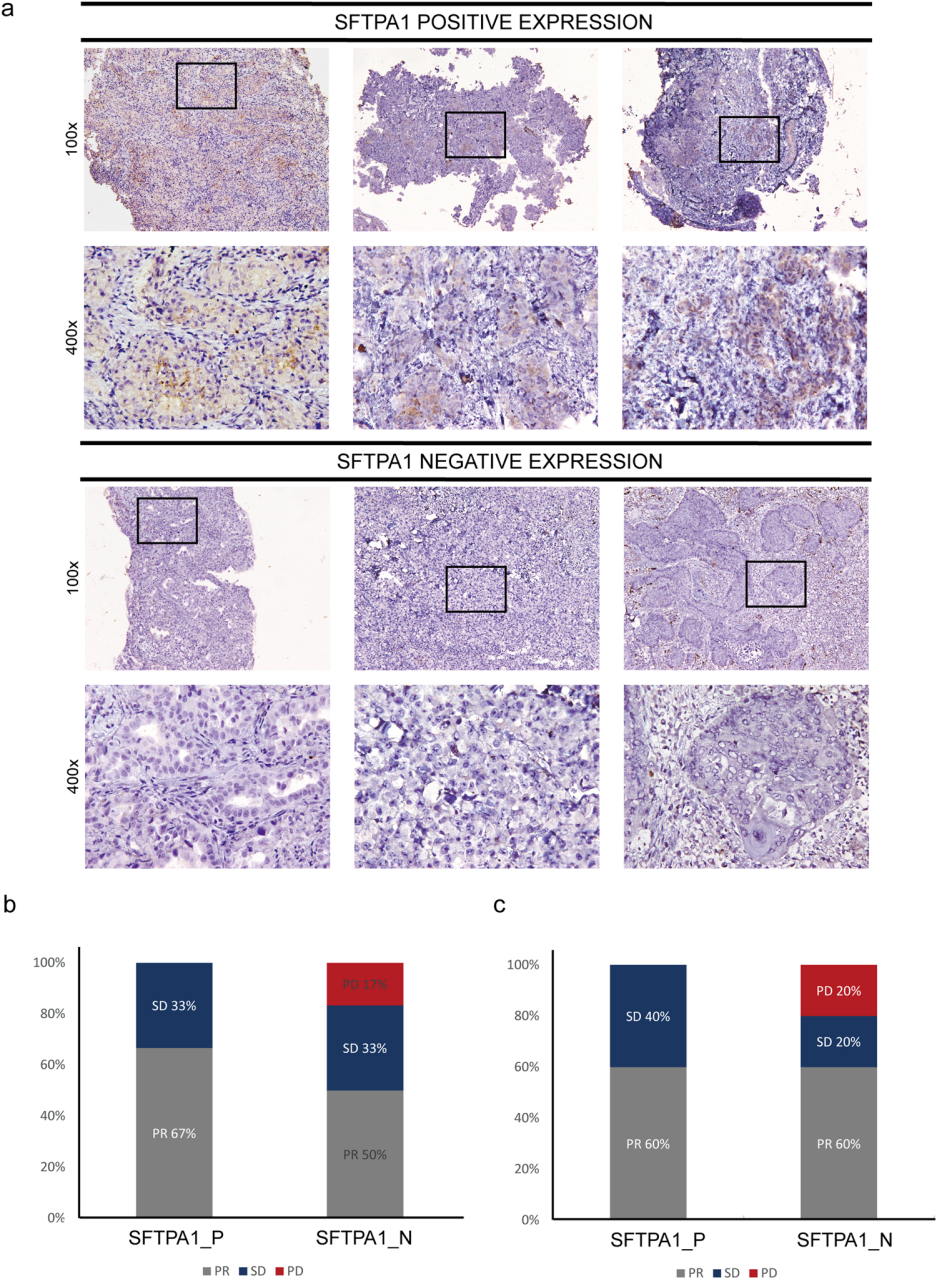
SFTPA1 expression and immunotherapy response. **a** SFTPA1 protein expression in lung cancer patients prior to immunotherapy by immunohistochemistry. All 14 patients were treated with a Pembrolizumab-based regimen. The positive SFTPA1 expression group (n = 6); negative SFTPA1 expression group (*n* = 6). **b** & **c** The immunotherapeutic efficacy of 12 NSCLC patients (**b**) and 10 lung adenocarcinoma patients (**c**) between SFTPA1 positive (SFTPA1_P) and the negative expression group (SFTPA1_N)

To identify the predictive power of SFTPA1 as an immunotherapy biomarker, we collected the tumor tissues of 12 lung cancer patients before treatment with a Pembrolizumab-based regimen. We categorized the 12 lung cancer patients into SFTPA1 positive/negative expression groups according to SFTPA1 protein expression (Fig. 8a). In this cohort, the pathological types included 10 adenocarcinomas and 2 squamous cell carcinomas. Efficacy was reported as the best overall response to treatment according to the Response Evaluation Criteria In Solid Tumors (RECIST) 1.1 standards. As shown in Fig. 8b, the positive SFTPA1 expression group exhibited a higher partial response (PR) rate than the negative group in all lung cancer patients (positive vs negative; PR: 67% vs 50%), and a lower progressive disease (PD) rate (0% vs 17%). Similar PD results (Fig. 8c) were obtained in adenocarcinomas patients (PD 0% vs 20%); however, the SD rate was higher (40% vs 20%), and the PR rate did not differ between the two groups (60% vs 60%) (Fig. 8c). Taken together, these results demonstrate that the immune-related gene, SFTPA1, displayed a strong potential to be a novel predictor of the response to immunotherapy.

## Discussion

SFTPA1 is a member of the C-type lectin subfamily, which has a unique collagen-like and spherical carbohydrate-binding domain. In addition, SFTPA1 plays an important role in the innate immune defense of the lungs. Previous studies have shown that the disruption of SFTPA1 homeostasis could induce both acute and chronic lung diseases, including respiratory distress syndrome and interstitial lung diseases [20]. Several studies have demonstrated that SFTPA is associated with the survival of patients with lung cancer [18, 21]. Only one study showed that SFTPA could regulate TAM polarization in lung cancer [18]. As a candidate immunomodulatory regulator, the role of SFTPA1 in the TIME and immunotherapy response deserves further investigation to understand the underlying mechanism.

We found some contradictions regarding the expression of SFTPA1 at both the mRNA and protein level. We presumed that there are two reasons for this discrepancy: (1) our sample size may have been too small, which may have introduced selection bias; and (2) Marcotte et al. [22] described that over approximately 60% of the changes at the protein level could not only be explained by the level of mRNA due to post-translational modifications, protein stability, and the relationship between the rates of the processes that produce and degrade them.

Of note, our Kaplan–Meier Plotter findings indicate the prognostic value of SFTPA1 and also suggest a functional heterogeneity of LUSC and LUAD. Some studies have shown that there are significant differences in the clinical stage, pathological stage, treatment, and the overall survival rate between LUAD and LUSC [23]. From a TIME perspective, Meng et al. [24] elucidated the differences in the immune microenvironment between squamous and nonsquamous NSCLC and their influence on patient prognosis. Similarly, our study identified different relationships between SFTPA1 and immune cell subsets in LUAD and LUSC. Unlike lung adenocarcinoma, high SFTPA1 expression was associated with an inactive immune microenvironment in LUSC, which may partially explain the different prognostic implications of SFTPA1. Considering that the results were consistent with the previous studies in LUAD, we focused on the pathological type of lung adenocarcinoma to investigate the molecular mechanisms of SFTPA1. Future pathway enrichment analyses revealed that SFTPA1 was closely correlated with the toll-like receptor (TLR) signaling pathway in the innate immune response. TLRs can recognize the pathogenic components or cellular debris and triggers a strong immunological response through cytokine secretion, thereby recruiting a large number of immune cells into the TME [25]. Recent studies suggest that TLR may represent an oncomir or a tumor suppressor depending on the tumor-infiltrating immune cells and cancer type. The study by Zhou et al. found that TLR5 overexpression was correlated with a better prognosis in NSCLC [26]. Furthermore, it was demonstrated that TLR3-dependent M1 induction was effective in murine lung cancer models [27]. Our results confirmed that SFTPA1 plays a vital role in the regulation of immune function via the TLR signaling pathway in LUAD.

It has been well-established that TIME is characterized by its high complexity and diversity. Macrophages are known to comprise the majority of the TME components and have extremely different effects, depending on their phenotype [28, 29]. There were typically two phenotypes: (1) classically activated M1 macrophages and (2) alternatively activated M2 macrophages. In general, the M1 macrophages likely provide protection against tumor growth, while M2 macrophages are likely promoting tumor growth, and the level of M2 macrophage infiltration is negatively correlated with the prognosis of patients. A rich CD4^+^ or CD8^+^ T cell density in the tumors was associated with a favorable clinical prognosis in lung cancer and several other types of tumors [30–32]. Yet, regulatory T (Treg) cells induce an immunosuppressive microenvironment, which is one of the major reasons for the efficacy of immunotherapy [33]. This finding is somewhat contradictory to our findings; however, the overall landscape indicates that the proportion of macrophages, CD8^+^ T cells, and CD4^+^ T cells were much larger than the regulatory T cells in LUAD. Moreover, recent studies have demonstrated that the TLR pathways plays an important role in macrophage polarization [34]. Thus, a reasonable conclusion can be drawn in that SFTPA1 could promote M1 macrophage polarization and CD4^+^/CD8^+^ T cell infiltration by activating the TLR signaling pathway.

It has been found that lncRNA ZFAS1 inhibits mitotic cycle 42 expression through regulating miR-590-3p in NSCLC cells [35]. Moreover, several studies have shown that has-miR-590-3p is involved in tumor development in several cancers, including ovarian cancers, colorectal cancer, and renal clear cancer [36–39]. Thus, has-miR-590-3p may function either as oncogenes or tumor suppressors. In our study, has-miR-590-3p is highly expressed in the tumor tissues and is inter‐regulated with lncRNAs to promote tumor development by targeting SFTPA1, which requires further research for verification. These results suggest that lung cancer patients with positive SFTPA1 expression were associated with a better response to the pembrolizumab-based regimen compared to the negative SFTPA1 expression group. We are currently collecting a larger dataset to further validate this finding.

Our findings demonstrate that SFTPA1 was up-regulated in the lung cancer tissues and was involved in the development and progression of lung adenocarcinoma through the regulation of various immune-related pathways. Our findings indicate that SFTPA1 may be a predictor of favorable prognosis and immunotherapeutic response for patients with lung adenocarcinoma. We believe that our findings will further help to establish novel preventive and therapeutic strategies for lung adenocarcinoma.

## Supplementary Information

Below is the link to the electronic supplementary material.

Figure S1. SFTPA1 expression pattern in human tissues. a. The level of mRNA expression in six candidate reference genes. b.The mRNA expression profile of SFTPA1 in human normal tissues using the GTExPOrtal database. c. SFTPA1 mRNA expression in human tumors using the publicly available Oncomine database (P < 0.01; fold-change > 2)
Supplementary file1 (TIF 16016 KB)

Figure S2. SFTPA1 co-expression genes and prognosis. a. Protein-protein interaction (PPI) network of SFTPA1 co-expression genes by STRING. b. There were 22 immune cell subpopulations in the SFTPA1-high expression group compared with the SFTPA1-low expression group in LUSC. (t test, P value Significant Codes: 0 ≤ *** < 0.001 ≤ ** < 0.01 ≤ * < 0.05). c. Venn diagram of immune-related genes and co-expression genes of SFTPA1 in TCGA LUAD. d. Heatmap of the correlation between the top 10 SFTPA1-related immune genes and immune cell infiltration landscape in TISIDB. e. Correlation between the expression of the top 10 immune SFTPA1-related genes and OS
Supplementary file2 (TIF 16216 KB)

Supplementary file3 (TSV 16 KB)

Supplementary file4 (TSV 11 KB)

Supplementary file5 (XLSX 813 KB)

## Data Availability

The data sets used and/or analyzed during the current study are available from the corresponding author upon reasonable request. The data that support the findings of this study are openly available in The Cancer Genome Atlas (TCGA) database (https://portal.gdc.cancer.gov/), Gene expression omnibus (GEO) database (http://www.ncbi.nlm.nih.gov/geo), and Genotype-Tissue Expression (GTEx) database (https://www.gtexportal.org/home/).
