# Supplementary figures and images for: SFTPA1 is a potential prognostic biomarker correlated with immune cell infiltration and response to immunotherapy in lung adenocarcinoma
Source: Cancer Immunol Immunother. 2021 Jun 28;71(2):399–415. doi: 10.1007/s00262-021-02995-4 (PMC8783894; doi:10.1007/s00262-021-02995-4)

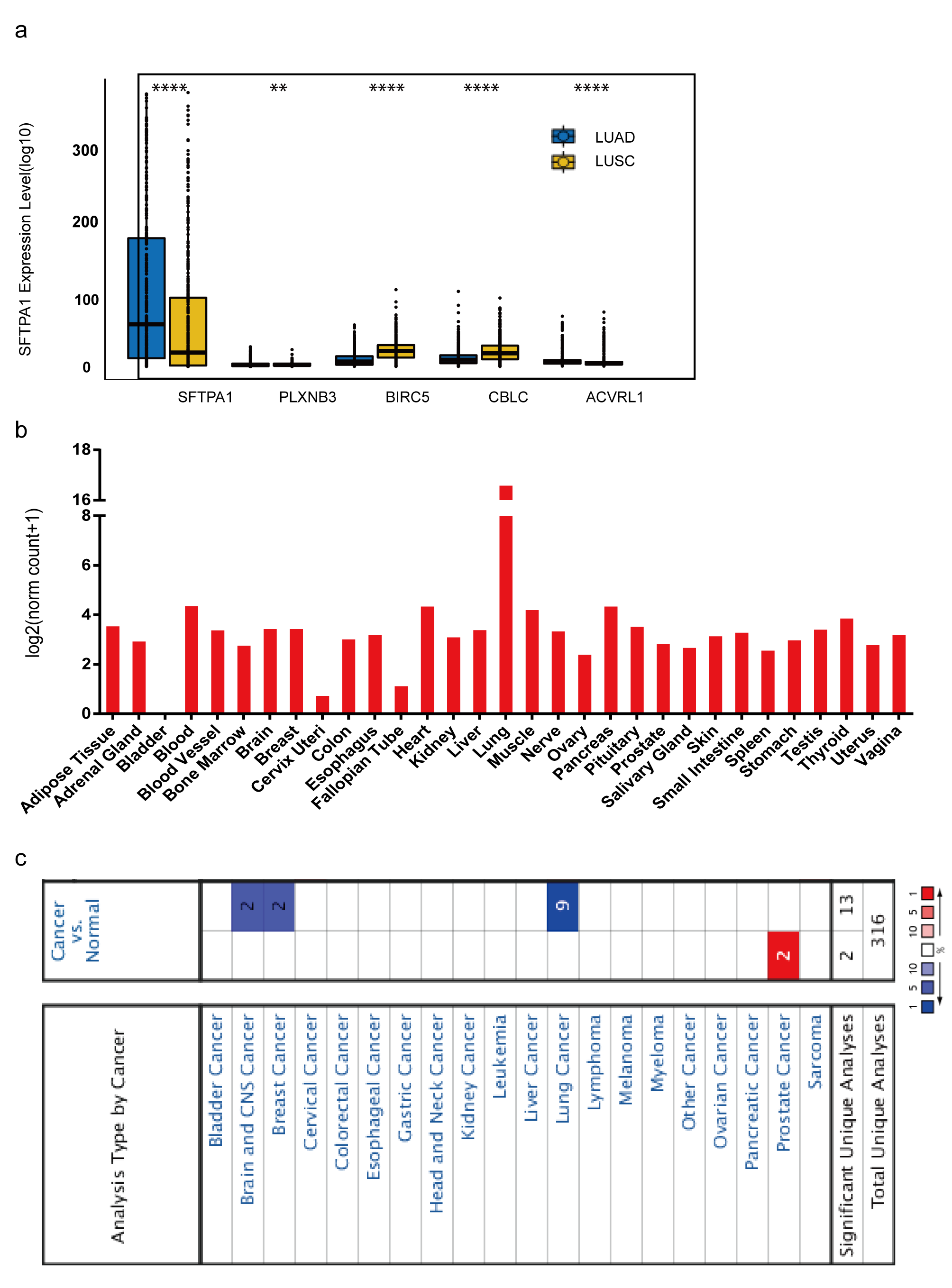

Supplement: Supplementary file 1 — Figure S1. SFTPA1 expression pattern in human tissues. a. The level of mRNA expression in six candidate reference genes. b.The mRNA expression profile of SFTPA1 in human normal tissues using the GTExPOrtal database. c. SFTPA1 mRNA expression in human tumors using the publicly available Oncomine database (P < 0.01; fold-change > 2) Supplementary file1 (TIF 16016 KB) [file 262_2021_2995_MOESM1_ESM.tif]

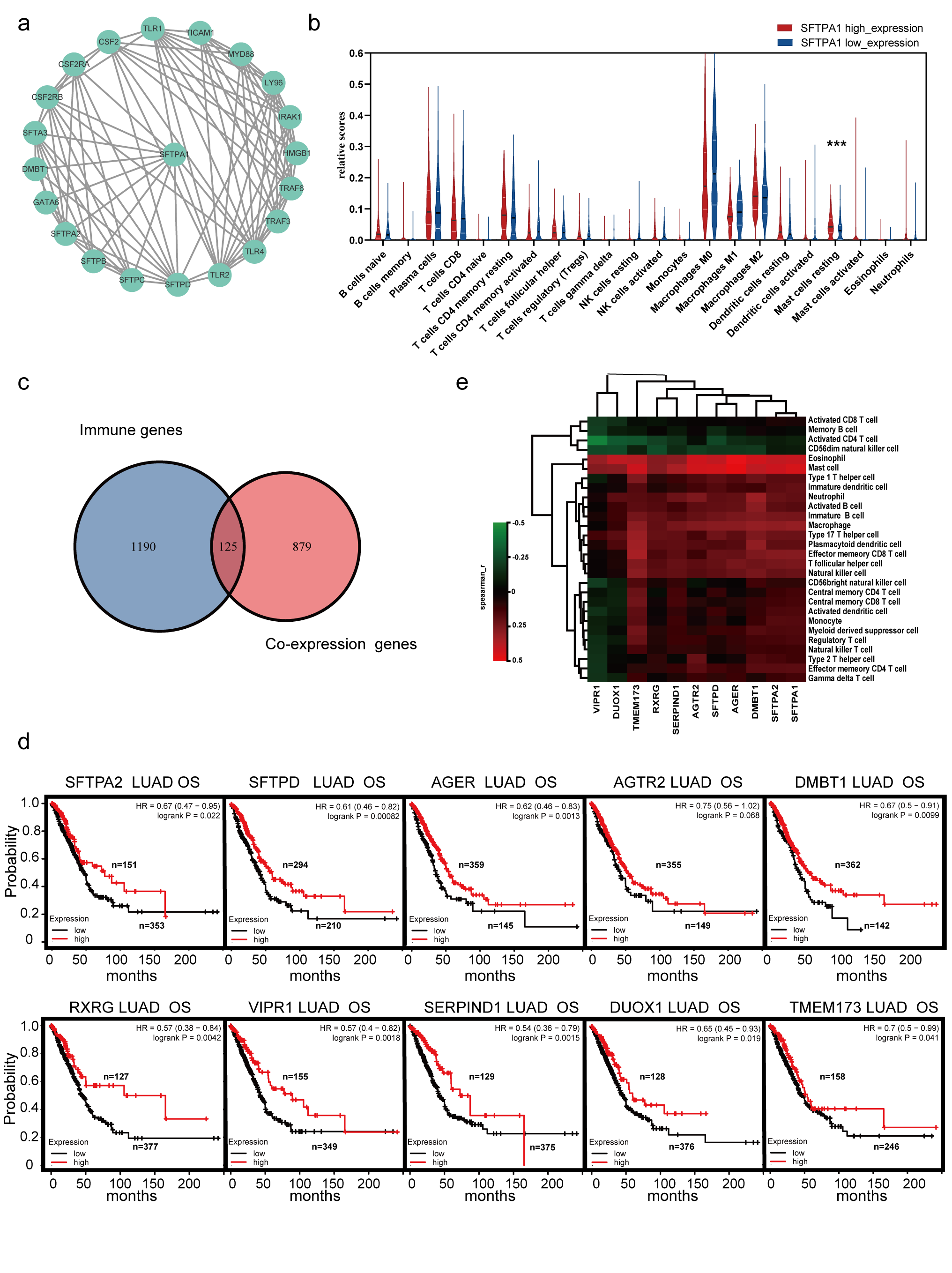

Supplement: Supplementary file 2 — Figure S2. SFTPA1 co-expression genes and prognosis. a. Protein-protein interaction (PPI) network of SFTPA1 co-expression genes by STRING. b. There were 22 immune cell subpopulations in the SFTPA1-high expression group compared with the SFTPA1-low expression group in LUSC. (t test, P value Significant Codes: 0 ≤ *** < 0.001 ≤ ** < 0.01 ≤ * < 0.05). c. Venn diagram of immune-related genes and co-expression genes of SFTPA1 in TCGA LUAD. d. Heatmap of the correlation between the top 10 SFTPA1-related immune genes and immune cell infiltration landscape in TISIDB. e. Correlation between the expression of the top 10 immune SFTPA1-related genes and OS Supplementary file2 (TIF 16216 KB) [file 262_2021_2995_MOESM2_ESM.tif]
